# Physicians’ attitudes in relation to end-of-life decisions in Neonatal Intensive Care Units: a national multicenter survey

**DOI:** 10.1186/s12910-020-00555-6

**Published:** 2020-11-23

**Authors:** Ilias Chatziioannidis, Zoi Iliodromiti, Theodora Boutsikou, Abraham Pouliakis, Evangelia Giougi, Rozeta Sokou, Takis Vidalis, Theodoros Xanthos, Cuttini Marina, Nicoletta Iacovidou

**Affiliations:** 1 2nd Neonatal Department and Neonatal Intensive Care Unit, Papageorgiou Hospital, Aristotle University of Thessaloniki, Thessaloníki, Greece; 2 Neonatal Department, School of Medicine, Aretaieio Hospital, National and Kapodistrian University of Athens, Athens, Greece; 3 2nd Department of Pathology, School of Medicine, “Attikon” University Hospital, National and Kapodistrian University of Athens, Athens, Greece; 4 Conseillère Direction Médicale CHR, Liege, Belgium; 5 Hellenic National Bioethics Commission, Athens, Greece; 6 European University of Cyprus, Nicosia, Cyprus; 7 Clinical Care and Management Innovation Research Area, Bambino Gesù Children’s Hospital, IRCCS, Rome, Italy

**Keywords:** End-of-life care, Withholding treatment, Withdrawing treatment, Attitude, Neonates, Neonatal Intensive Care Units

## Abstract

**Background:**

End-of-life decisions for neonates with adverse prognosis are controversial and raise ethical and legal issues. In Greece, data on physicians’ profiles, motivation, values and attitudes underlying such decisions and the correlation with their background are scarce. The aim was to investigate neonatologists' attitudes in Neonatal Intensive Care Units and correlate them with self-reported practices of end-of-life decisions and with their background data.

**Methods:**

A structured questionnaire was distributed to all 28 Neonatal Intensive Care Units in Greece. One hundred and sixty two out of 260 eligible physicians answered anonymously the questionnaire (response rate 66%). Demographic and professional characteristics, self-reported practices and opinions were included in the questionnaire, along with a questionnaire of 12 items measuring physicians’ attitude and views ranging from value of life to quality of life approach (scale 1–5).

**Results:**

Continuation of treatment in neonates with adverse prognosis without adding further therapeutic interventions was the most commonly reported EoL practice, when compared to withdrawal of mechanical ventilation. Physicians with a high attitude score (indicative of value of quality-of-life) were more likely to limit, while those with a low score (indicative of value of sanctity-of-life) were more likely for continuation of intensive care. Physicians’ educational level (*p*:0.097), involvement in research (*p*:0.093), religion (*p*:0.024) and position on the existing legal framework (*p* < 0.001) were factors that affected the attitude score.

**Conclusions:**

Physicians presented with varying end-of-life practices. Limiting interventions in neonates with poor prognosis was strongly related to their attitudes. The most important predictors for physicians' attitudes were religiousness and belief for Greek legal system reform.

## Background

Advances in neonatology over the last decades, have significantly improved survival of premature infants [1-3]. As long-term morbidity of the survivors hasn't improved proportionately, unconditional invasive intensive care raises ethical dilemmas [3]. End of life decisions (EoLDs) for severely ill newborns, though aiming at alleviating suffering and pain, they still are a difficult and highly emotional task, often controversial from medical, ethical and legal viewpoints [4, 5].

EoLDs focus on managing the late stage of life and comprise a key component of the palliative care in the Neonatal Intensive Care Unit (NICU) population [6]. Medical ethics focus on advocating the patient’s best interests, by not causing harm, ensuring justice, and shielding the patient’s right (represented by proxy—the parents in the case of infants) to refuse or choose his/her treatment [7]. Dilemmas regarding the intrinsic value and importance of life quality, in cases of uncertain prognosis, make medical decisions extremely burdensome [2, 3, 8, 9]. Quality of life is related to a person’s wellbeing (i.e. emotional, social, physical) or mental capability or simply the ability to perform ordinary tasks, although opinions differ [4]. EoLDs may vary between sustaining life at any cost, regardless the prospect of serious morbidities (sanctity of life approach), to limiting intensive care provision (quality of life approach) [1, 10].

Εnd-of-Life decisions refer to decisions that a Neonatologist may be called upon to take for neonates with severe brain injury and poor neurologic prognosis, neonates with serious congenital malformations or untreatable genetic conditions incompatible with life, marginally viable neonates at 22^+0/6^–23^+6^ weeks gestation, and terminally ill neonates at a non-reversible state [4, 11]. Quality of provided antenatal/prenatal care and improved prematurity survival rates, underline the necessity of empirical data collection for the implementation of specific policies in the NICUs.

Data in the literature showed that EoLDs are associated with physicians’ demographic and professional factors such as age, gender, professional experience, position, religion and religiousness [1, 3]. Decisions concerning terminally ill newborns are influenced by personal moral values and attitudes of both physicians and parents. A study across several European countries showed wide variations of medical policies across countries of different social/cultural background and legislation status [1]. There is no consensus on potential choices and factors to be considered, especially in countries like Greece, where specific legal provisions and/or medical ethics guidelines for NICU patients does not exist. Around 62–93% of NICU deaths worldwide follow EoLDs as to withhold and primarily to withdraw treatment, thus affecting infant mortality [12–15]. Consequently, EoLDs should be considered in the evaluation of NICUs’ treatment outcomes, as for survival in respect to the NICU’s morbidity rate [16].

Recent literature provides valuable data on physicians’ opinions, attitudes and end of life practices, particularly regarding long term morbidity of extremely premature infants [2, 17, 18]. EoL decisions are difficult to implement, therefore comparison with neonatologists’ applied practices in other countries is always useful [9, 19, 20]. There are two studies on healthcare professionals’ attitudes in Greek NICUs, referring to data from the same survey collected a decade ago [21, 22]. The present nationwide multicenter survey was conducted to estimate, on a more representative basis, and evaluate possible differences in EoL decisions through the elapsed time.

The objectives of the study were: (1) to assess the frequency with which physicians are involved in EoLDs, (2) to investigate the type of applied practices, (3) to investigate physicians’ attitudes towards value of life, and (4) to examine the impact of personal and professional characteristics on their attitudes.

## Methods

### Data collection

We formed a questionnaire based on that of the EURONIC study [10] (with the kind permission of the authors), with the addition of some further questions which we estimated that they would reflect our national values. The study questionnaire is provided as Additional file. To confirm agreement for equivalence and validation purposes, the questionnaire was translated from English to Greek and then again to English, for the final comparison of the original and translated version.

### Participants

An anonymous structured questionnaire was sent to all Neonatologists employed in the 28 NICUs across the country with a prepaid return envelope. A cover letter explaining the purpose of the study was also included; a reminder after 4 weeks was sent to those who had not responded promptly. Data was collected between September 2018 and January 2019

### Questionnaire

The questionnaire included 16 questions and consisted of four sections:

*Section 1 (Q 1–13)* included information on professional and demographic characteristics. Participant's professional group, job rank, qualifications/education, employment contract, working experience in the field, working hours per week, daily duties, participation to follow up and involvement in research programs, gender, age, parenthood, religious background and importance of religion were collected.

*Section 2 (Q 14–15) *included self-reported practices of EoLDs in certain neonatal groups (those with severe neurological prognosis, at end stage, with poor prognosis and extreme prematurity). EoLDs included withdrawing treatment, avoiding emergency treatment, withholding treatment, continuation of ongoing treatment without adding further therapeutic interventions, withdrawal of mechanical support and administration of drugs even at the risk of respiratory depression and death as reported by Cuttini et al. [10].

*Section 3 (Q 16) *included personal views regarding Greek law reform.

In Section 4 (Q 16) data referred to attitudes on limitation or continuation of intensive care The 12-item questionnaire indexed scale, was used to assess attitudes by respondent’s agreement, on a 5-point Likert scale (from 1 “strongly agree” to 5 “strongly disagree”) developed by Rebagliato et al. [1]

The research protocol was approved by the Medical Research Ethics Committee, Aretaieion Hospital, National and Kapodistrian University of Athens (Ref. No. 112/13-02-19).

### Statistical analysis

All questionnaires were collected and coded in Microsoft Excel for subsequent analysis. Data analysis was performed via the SAS for Windows 9.4 software platform (SAS Institute Inc., NC, USA). Statistical analysis was also performed with SPSS 17.0v for Windows (SPSS Inc., Chicago, Ill, USA). *P* value < 0.05 was considered statistically significant.

Numerical data was expressed as mean ± standard deviation, and for completeness as median, interquartile range, minimum and maximum values; for categorical data, the relevant percentages within individual groups were used. For numerical data with non-normal distribution, non-parametric tests were used, specifically the Kruskal–Wallis test; for the comparison of categorical variables for differences between groups frequencies, χ^2^ test was used.

Factor analysis was used to identify the underlying dimension of the 12 attitude items. In the 12-item attitude questionnaire, participants were asked to grade on a scale of 1 to 5 their attitude for the value of life (see Additional file 1). The reliability of the complete test was tested by Cronbach’s alpha (standardized) [23] and was calculated at 68.7%. In order to reveal correlation between items of the questionnaire Spearman correlation coefficient was used. Subsequent factor analysis revealed that a single factor could explain 69.9% of the total variance. The items contributing to this factor according to the highest loadings were questions (attitude items) 1, 2, 3, 6, 7 and 8 and had an acceptable Cronbach’s reliability α = 78.0%. A score was created based on the sums of these items after weighting by their loadings (i.e. each item was multiplied by the loading and subsequently all products were added); this score is subsequently called attitude score. A low attitude score was indicative of medical decisions towards sustaining life at any cost, despite potential severe morbidities (sanctity of life approach) while a high attitude score towards withdrawing intensive care in cases of poor prediction (quality of life approach). Moreover, the score was normalized in order to produce comparative results to other studies [1] between 0 (indicative for sanctity for life approach) and 10 indicative for quality of life approach. Reported EoL practices were evaluated in relation to the attitude score and a binary logistic regression model predicting a positive response on the basis of attitude score was subsequently constructed.

We used factor analysis in the preset study, because it creates a theoretical model of latent factors that cause the observed variables (i.e. the questionnaire items). The main factor of this analysis was used as the attitude score and explains very high percentage of the responses, thus it produced a single number that is related to the participants’ behavior. A second reason for preferring factor analysis instead of principal component analysis (PCA) is the fact that this technique had already been used in another study, thus this report produces compatible and comparable results.

A univariate analysis was used to identify the variables associated with a physician's attitude score, with the score as the dependent variable. Independent variables included demographic and professional characteristics of the respondents. The variables retained in the final multivariate analysis (by a generalized linear model) were correlated with the attitude score at *p* < 0.10, while variables not significantly related to the attitude score were removed from the model. In all analyses, data were based on valid responses for each group or subgroup, since not all respondents answered all questions.

## Results

The study inquired participation of all 28 NICUs across country; Finally 23 NICUs participated, and all 5 NICUs that did not participate in the study were excluded. A total of 156 completed questionnaires were returned from 236 eligible employees with an overall response rate of 66%. The percentage average for non-response to individual questions was 2.7% (highest non-response in working years, 24.36%) (Table 1).

**Table 1:** Characteristics of study participants

Occupational group	N (%, 95%CI), N = 156
*Gender*	
Male	30 (19.2, 13.0–25.4)
Female	126 (80.8, 74.6–87.0)
*Age (years)*	
< 40	45 (28.9, 21.7–36.0)
> 40	111 (71.1, 64.0–78.3)
*Having had children (parenthood)*	
No	33 (23.2, 16.3–30.2)
Yes	109 (76.8, 69.8–83.7)
*Religious background*	
Christian orthodox	143 (94.1, 90.3–97.8)
Catholic	1 (0.7, 0.0–3.6)
Protestant	2 (1.3, 0.2–4.7)
Atheist	6 (3.9, 1.5–8.4)
*Religion importance*	
Important	71 (46.4. 38.5–54.3)
Quite or not important	82 (53.6, 45.7–61.5)
*Daily duties*^a^	
Yes	112 (74.2, 37.2–81.2)
No	39 (25.8, 18.5–32.8)
*Years of employment*^a^	
< 6	77 (49.4, 41.5–57.2)
6–15	39 (25.0. 18.2–31.8)
> 15	40 (25.6, 18.8–32.5)
*Working hours (per week)*^a^	
< 40	38 (24.4, 17.6–31.1)
> 40	118 (75.6, 68.9–82.4)
*Education*	
Graduate from medical school	86 (55.5, 47.7–63.3)
M.Sc. and/or Ph.D.	69 (44.5, 36.7–52.3)
*Follow up in outpatient clinic*	
Yes	121 (79.1, 72.6–85.5)
No	32 (20.9, 14.5–27.4)
*Employment contract*^a^	
Tenure	82 (54.3, 46.4–62.3)
Non-permanent	69 (45.7, 37.7–53.6)
*Involvement in research*^b^	
Yes	81 (52.6, 44.7–60.5)
No	73 (47.4, 39.5–55.3)
*Physician’s rank*	
Fellow resident	58 (37.1, 29.5–44.7)
Consultant	32 (20.5, 14.2–26.8)
Research associate	57 (36.5, 28.9–44.1)
Neonatologist (part time)	9 (5.7, 2.1–9.3)
*Position on existing legal framework*^c^	
Should change	104 (73.3, 66.0–80.5)
Should not change	30 (21.1, 14.4–27.8)
Not relevant	8 (5.6, 1.8–9.4)

^a^In the NICU

^b^At least one research project in the previous year

^c^In Greece, euthanasia is strictly prohibited in accordance to Article 300 of Penal Code and Article 29 of the Code of Medical Ethics. As for neonates there is no specific report on treatment limitation or palliative care

### Reported practices in NICUs

**Fig. 1:**
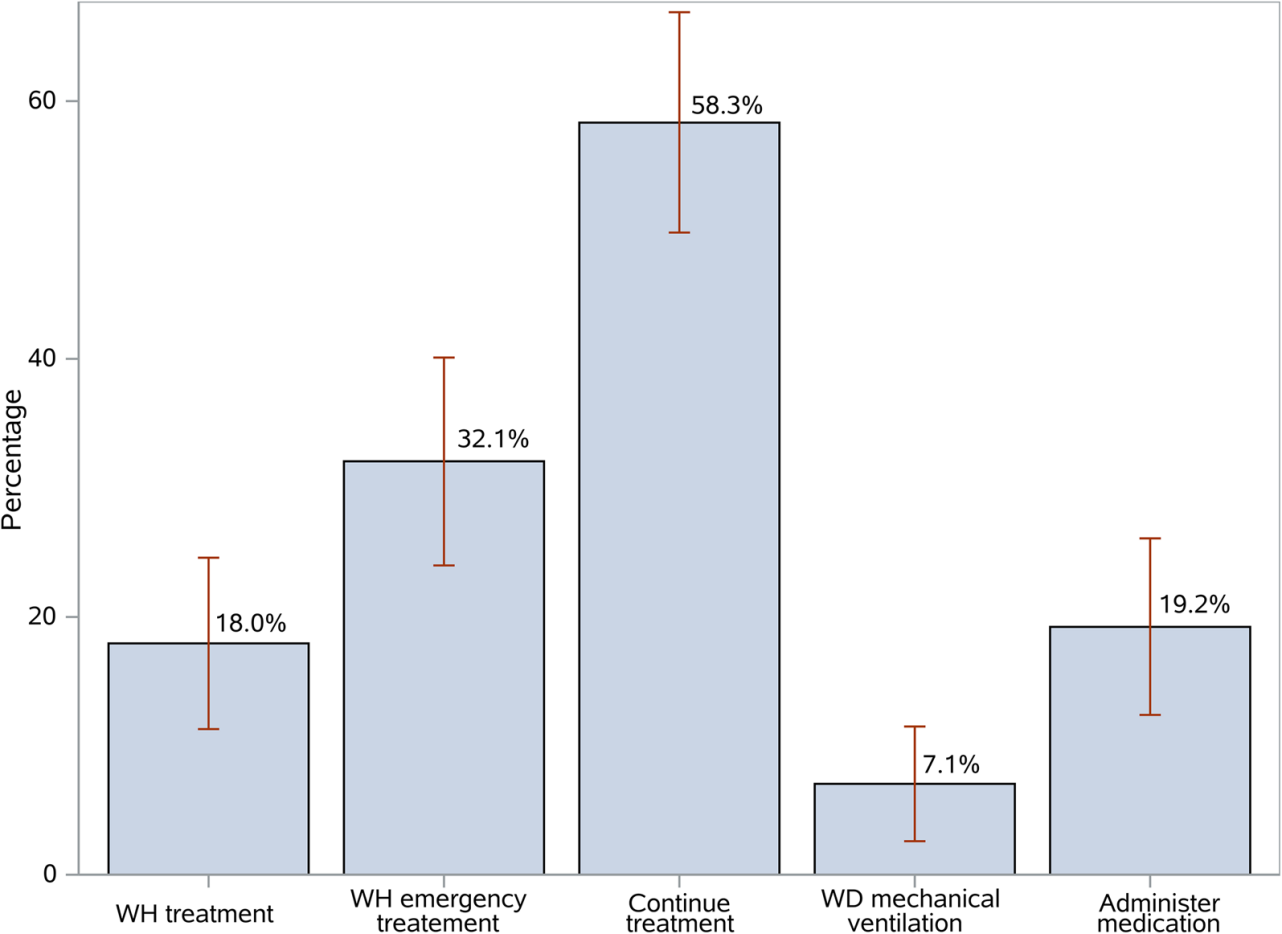
Proportions and 95% confidence limits for the physicians that responded positively on the questions for EoL practices. WH: Withhold, WD: withdraw. The five options for EoL practices in details: WH treatment: withhold treatment (resuscitation at birth, mechanical ventilation), WH Emergency treatment: Avoid Emergency Treatment (CPR), Continue treatment: continue with ongoing treatment without adding further therapeutic interventions, WD mechanical ventilation: Mechanical ventilation withdrawal and Administer medication: Administration of sedatives and/or analgesic drugs to comfort the neonate, even at risk of respiratory depression and death

The proportion of physicians having ever decided to limit intensive care as an EoL practice is shown in Fig. 1. The most common EoL practice was to continue with ongoing treatment without adding further treatment (58.3%), while the least common was mechanical ventilation withdrawal (7.1%).

**Fig. 2:**
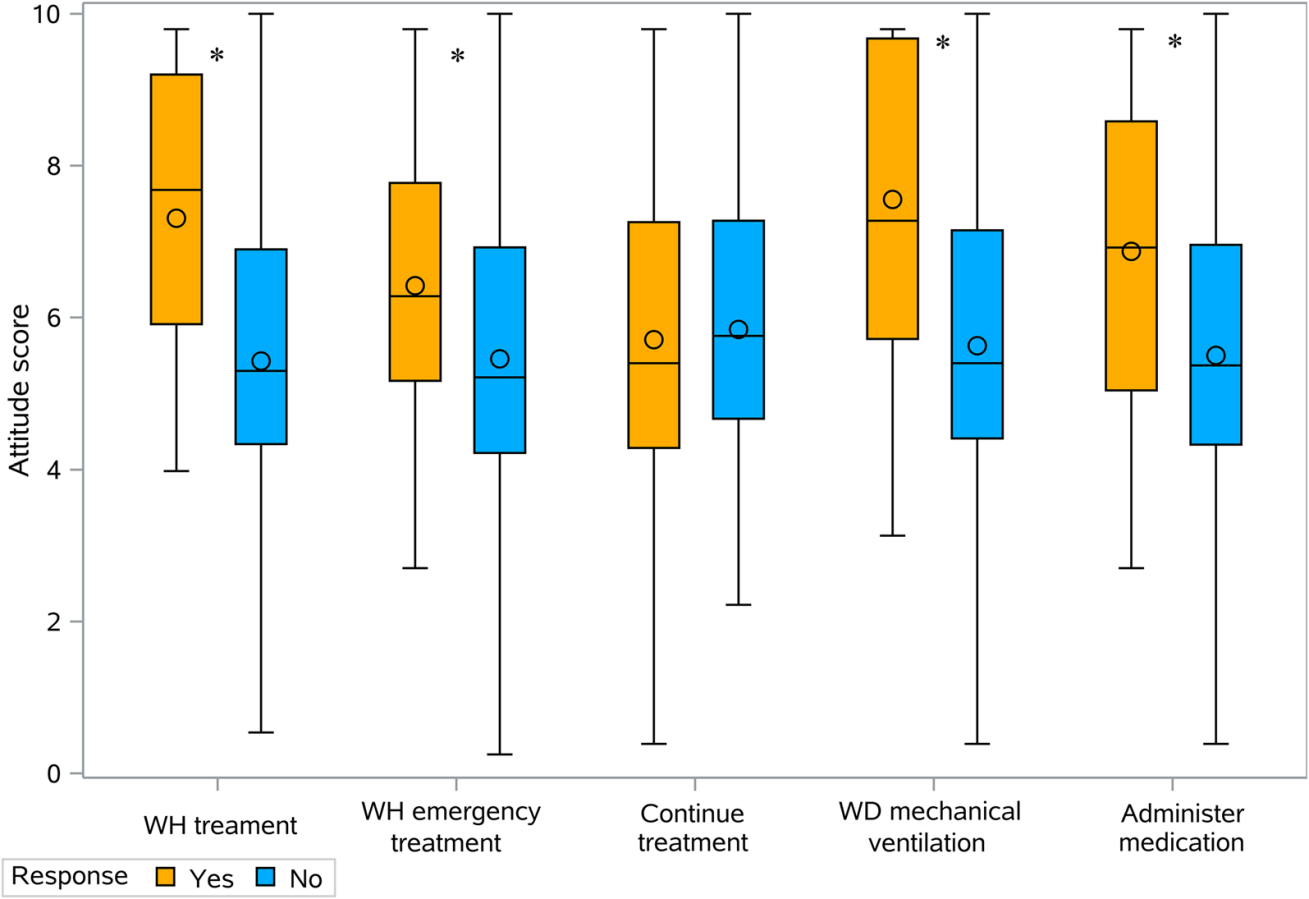
Attitude score indexed by 12-item Questionnaire exploring EoL Practices. Each pair of box and whisker plots is related to a single practice. Ends of whiskers show minimum and maximum values, the lower and upper part of the boxes refer to 25th–75th percentile respectively, the line inside boxes the median value and the symbol within each box the mean value (outliers excluded). The asterisk symbol shows statistically significant difference in the attitude score between a positive and negative responses. *WH* Withhold, *WD* withdraw

The attitude score indexed by a 12-item questionnaire was significantly higher in physicians reporting setting limits in intensive care for all EoL practices other than continuation of treatment without adding further treatment options (Fig. 2, Table 2).

**Table 2:** Attitude score indexed by 12-item questionnaire exploring EoL practices according to responses

	N	Mean ± SD	Range	Median	Q25–Q75	*p*
WH treatment						
Yes	28	7.3 ± 5.4	0.4–9.8	7.7	5.9–9.2	* < 0.0001*
No	128	5.4 ± 2.0	0–10	5.3	5.3–6.9	
WH emergency treatment						
Yes	50	6.4 ± 2.1	0.4–9.8	6.3	5.0–7.8	*0.0039*
No	106	5.5 ± 2.1	0–10	5.2	4.0–6.9	
Continue treatment						
Yes	91	5.7 ± 2.2	0.4–9.8	5.4	4.0–7.3	0.5003
No	65	5.8 ± 2.2	0–10	5.8	5.8–7.3	
WD mechanical ventilation						
Yes	11	7.6 ± 2.3	3.1–9.8	7.3	6.0–9.7	*0.0061*
No	145	5.6 ± 2.1	0–10	5.4	4.0–7.2	
Administer medication						
Yes	30	6.9 ± 2.0	2.7–9.8	6.9	5.0–8.6	*0.0029*
No	126	5.5 ± 2.1	0–10	5.4	4.0–7.0	

*WH* withhold, *WD* withdraw, *administer medication* administration of sedatives and/or analgesic drugs, *SD* standard deviation, *Q25–Q75* quantile 25–75

### Physicians’ characteristics and attitude score

In univariate analysis gender, age, parenthood, daily duties in the NICU, years of employment, working hours per week, involvement in follow up, employment contract and rank did not seem to be associated with the attitude score (see Additional file 2). Physicians who considered religion as non-important, had higher educational level, participated in research projects and were positive on changing the current law framework, had statistically significant higher attitude scores. The multivariate linear model analysis showed-after adjustment for the individual participating NICUs- that the attitude score was mainly affected by religion importance (*p* = 0.0989, 0.6 ± 0.4 units decrease of the attitude score for an opinion that religion is important) and belief on changing the current law framework (*p* = 0.0022, 1.5 ± 0.4 units increase of the attitude score for an opinion that the law framework requires reform) for EoL care. These results are compatible with the univariate analysis as the difference in the mean attitude score was 0.9 units lower for those who considered religion is important and 1.6 units higher for those who considered that the legal system requires reform.

### Association of doctors’ characteristics with EoL practices

Association of physicians’ characteristics and reported practices is shown in Table 3. Gender was found important for the administration of medication; specifically, males had 3.3 higher odds (95% CI 1.3–7.9, *p* = 0.007) *vs.* women to administer drugs for sedation (men 36.7% vs. women 15%). Regarding age, younger doctors were less likely to withhold emergency treatment and simultaneously less likely to withdraw mechanical ventilation (below 40 years 16% would avoid CPR, while above 40 years 34.9%). Moreover, all 11 doctors that “would remove mechanical ventilation” were > 40 years. Additionally, doctors with < 6 years employment experience were less likely to withhold emergency treatment (OR: 0.4, 95% CI 0.2–0.9, *p* = 0.0219). Participation in a research project had a significantly higher impact (OR: 10.1) in deciding to withdraw mechanical ventilation (10 out of the 11–91%—of doctors that would remove mechanical ventilation had participated in a research program, while only 50% from doctors who would not remove mechanical ventilation had involvement in research project experience). The most influential parameter on EoL practices was the belief in legal system reform, with physicians’ in favor, having considerably higher odds to have a positive response in most of the EoL decision options (withhold treatment or emergency treatment and withdraw mechanical ventilation). Parenthood, religion importance, daily duties in the NICU, working hours, education level, involvement in follow-up, employment contract (tenure or not permanent) and rank (certified Neonatologist or Fellow) were not found to influence the EoL decisions of the doctors.

**Table 3:** Association of physicians’ characteristics with EoL practices

	N	WH treatment (N_Yes_ = 28)	WH emergency treatment (N_Yes_ = 50)	Continue treatment (N_Yes_ = 91)	WD mechanical ventilation (N_Yes_ = 11)	Administer medication (N_Yes_ = 30)
Gender						
Male	30	1.2 (0.4–3.2)	0.7 (0.3–1.8)	1.3 (0.6–2.9)	1.6 (0.4–6.6)	*3.3 (1.3–7.9)*
Female	126	0.7446	0.4819	0.5365	0.4827	*0.007*
Age						
< 40	45	0.4 (0.1–1.1)	*0.4 (0.2–0.8)*	0.6 (0.3–1.2)	*	2.2 (1–5.1)
≥ 40	111	0.0605	*0.015*	0.1276	*0.0285*	0.0513
Having had children						
No	33	1 (0.4–2.7)	0.7 (0.3–1.6)	1.3 (0.6–3)	0.7 (0.1–3.5)	1.3 (0.5–3.4)
Yes	109	0.9827	0.3654	0.4902	0.6792	0.5344
Religion importance						
Important	71	0.6 (0.2–1.4)	0.8 (0.4–1.5)	1.4 (0.7–2.7)	0.6 (0.2–2.3)	0.5 (0.2–1.2)
Quite or not important	82	0.2095	0.4465	0.2866	0.4882	0.1093
Daily duties						
Yes	112	1 (0.4–2.6)	0.8 (0.4–1.7)	0.9 (0.4–1.9)	0.4 (0.1–1.3)	0.6 (0.2–1.4)
No	39	0.9897	0.5934	0.7748	0.1225	0.2361
Years of employment						
< 6	77	0.6 (0.3–1.4)	*0.4 (0.2–0.9)*	0.7 (0.3–1.3)	1.3 (0.4–4.3)	1 (0.5–2.3)
≥ 6	79	0.2392	*0.0219*	0.2033	0.7212	0.9377
Years of employment						
< 15	115	1.1 (0.4–2.8)	1.2 (0.5–2.6)	0.7 (0.3–1.4)	3.8 (0.5–30.7)	1 (0.4–2.4)
≥ 15	41	0.8649	0.6565	0.2553	0.1791	0.9575
Working hours (per week)						
< 40	38	0.6 (0.2–1.8)	1.3 (0.6–2.9)	1.5 (0.7–3.2)	1.2 (0.3–4.7)	0.9 (0.4–2.4)
≥ 40	118	0.3762	0.4668	0.2837	0.8154	0.8842
Education						
Graduate from medical school	86	0.5 (0.2–1.2)	0.6 (0.3–1.3)	0.9 (0.5–1.8)	0.6 (0.2–2.2)	1.1 (0.5–2.4)
M.Sc. and/or Ph.D.	69	0.1375	0.1958	0.8721	0.4874	0.8846
Follow up in outpatient clinic						
Yes	121	0.8 (0.3–2)	0.9 (0.4–2.1)	0.7 (0.3–1.5)	0.7 (0.2–2.7)	2.8 (0.8–9.8)
No	32	0.5565	0.8182	0.3364	0.5905	0.1011
Employment contract						
Non-permanent	69	0.6 (0.3–1.4)	1 (0.5–2)	0.7 (0.4–1.3)	1 (0.3–3.4)	1.1 (0.5–2.6)
Tenure	82	0.2401	0.9578	0.2873	0.9867	0.7563
Involvement in research						
Yes	81	1.3 (0.5–2.9)	0.8 (0.4–1.6)	0.5 (0.3–1)	10.1 (1.3–81.3)	1.6 (0.7–3.7)
No	73	0.5944	0.5391	0.0576	0.0083	0.2569
Physician’s rank						
Fellow	58	1 (0.4–2.3)	0.5 (0.2–1)	0.6 (0.3–1.3)	1.9 (0.6–6.7)	1.4 (0.6–3.3)
Specialized Neonatologist	89	0.9837	0.0562	0.2002	0.2871	0.4015
Position on existing legal framework						
Should change	104	*13 (1.7–99.2)*	*4.8 (1.7–13.4)*	0.9 (0.4–1.9)	**	2.4 (0.8–7.5)
Should not change or not relevant	38	*0.002*	*0.0012*	0.7616	*0.0369*	0.1192

For each comparison the odds ratio, 95% confidence interval and *p *value are depicted. In each row total number of cases for group options (as N) and in each column positive answers in the specific question (as N_Yes_) are reported. There was only one positive response in the reported practice C6; thus, no statistical comparisons were performed

*WH* withhold, *WD* withdraw, *administer medication* administration of sedatives and/or analgesic drugs

^*^Not applicable all participants that declared yes had age ≥ 40

^**^Not applicable all participants that declared no or not relevant responded no in this question

## Discussion

The present study showed that physicians were involved in various EoL practices. Interventions in neonates were found strongly related to their attitudes. A high attitude score is indicative of the quality of life approach (otherwise not sustain life at any cost) while a low score agrees with the approach of the absolute value or sanctity of life approach (otherwise sustain life despite potential severe morbidities). EoL practices differ in association to the attitude score, with a higher attitude score favoring quality of life and limitation of intensive care at a more aggressive model of approach, while a lower score favors sustaining life by continuation of treatment at a more conservative model of approach; additionally, religion importance and position on legal framework were the main determinants of the attitude score.

Daglas et al. [21, 22] study on the attitude of Greek healthcare professionals in NICUs reported a mean attitude score of 3.1 indicative that Greek healthcare professionals tended to support the value of human life. Contrarily, our study reports an average attitude score of 5.8 indicative of a shift towards the value for quality of life. This difference could be attributed to various factors: a) different sample composition; in the above mentioned study 251 healthcare professionals participated and only 71 were physicians, while our study is focused on physicians’ opinion (156 doctors’ respondents), a more than the double the size sample, b) different approach to calculate the attitude score: Daglas et al. study calculated the attitude score as the sum of each participant’s answers to the 7 questions that were found important; in our study 6 items of the questionnaire that were found important were weighted by their loadings before extracting the final attitude score, and finally, c) our study data were collected 10 years after Daglas et al. study (from May 2009 to May 2011). During these years Greece faced a severe economic recession with many burdens imposed on the population on social and cultural level. Due to the fact that during the time elapsed physicians have experienced survivors with major disability born at the limit of viability or had faced major events at birth, we could speculate that a redefinition of their personal beliefs and way of thinking has occurred.

Recent data in the literature shows increased frequency of withholding or withdrawing treatment [24, 25]. EURONIC multicentre ethical research study and subsequent studies showed that withholding or withdrawing treatment is a common practice in several European NICUs and worldwide [1, 14, 26–29] One out of three till five physicians decided withholding of treatment, while one out of ten decided withdrawing of treatment: these ratios are lower than these reported in other European countries and much similar to that in South Mediterranean countries. The absence of a clear distinction between treatment withdrawal and assisted dying is a key factor in differing practices with treatment withholding [30]. Not all professionals accept that withholding and withdrawing treatment are morally equivalent. Moreover, there are arguments that withdrawal of treatment leads to assisted dying [9]. Supportive to this pattern is the fact that only one out of five physicians chose to administer drugs even at risk of death lower to recent studies [31]. Physicians seem to accept non-treatment decisions (as to withhold or withdraw treatment) and administration of drugs even at risk of death, clearly outside Greek legal framework (article 300 of the Penal Code and article 29 of the Code of Medical Ethics refers to euthanasia strictly prohibiting the act).

Lack of legal framework for NICUs various legal constraints, of firm policies even within NICUs, psychological support [31], avoidance of approaching in public ethical issues that raise dilemmas lead to a more conservative EoL approach by physicians.

Decision-making is a multifactorial task, dependent on knowledge, relationships, life experiences and subjective approach as attitude for life issues [17]. The majority of studies on end-of-life care in severely ill newborns describe physicians’ attitudes and not their implemented practices [28]. Attitude score relates to quality of life and limitation of intensive care or sustaining life and continuation of treatment and is well established by findings in the EURONIC project [1]. In the present study we showed that physicians’ EoL reported practices were associated with the attitude score; physicians with a higher attitude score showed a tendency for a positive answer, while those with lower attitude score were prone to non-participating. Attitude scores did not differ significantly only when physicians “continued ongoing treatment without adding other therapeutic interventions”. The higher the physicians’ attitude score, the more probable action was to intervene in EoL practices, showing a clear preference to limit life without hope, while a lower score was oriented towards prolonging life. Differences concerning the attitude among physicians in other studies could be attributed to the different cultural and social background, in addition to physicians’ characteristics [12–15, 30, 32]. Rebagliato et al. [1] reported that after controlling for confounders, country of origin remained a significant predictor of physicians’ attitude score and practices suggestive of social and cultural factors.

Physicians’ characteristics with a statistically different attitude score were education, involvement in research, religion importance and position on changing the current legal framework; in the final analysis only religiousness and law change belief remained as the main predictors of the attitude score. Religious beliefs are highly influential factors when making life and death decisions for infants, both for the physicians and parents [33].

The effect of physician’s characteristics and beliefs shows that clinical factors, legislation and social culture are not the only predictors for ethical decisions. Indeed, as ethical decisions derive from personal moral principles and values, parents have the right to be informed on physicians’ attitudes and personal beliefs [20, 33].

Additionally to previous research [1, 31], male gender, younger age, less time of experience, participation in research projects and most importantly law change belief were strong indicators for EoL practices towards limiting intensive care.

### Strengths and limitations

The sample which is national representative, the prospective design and detailed data collection are strong points of the present study. Since objectives were met, it may provide a ground for generalization. There are several limitations to be underlined. First, the attitude score was derived through factorial analysis by selecting the statements that showed a high correlation and internal consistency, as well as content validity with attitudes towards life value and support. Second, the subjective nature of the study cannot exclude underreporting of EoLDs, despite protection of anonymity and confidentiality. Third, we did not collect demographic data for the non participated physicians and finally 5 out of 28 NICUs did not participate in the study and were excluded.

### Implications

EoLDs and clinical indications for therapeutic limitations vary considerably between countries. As EoLDs are a key-factor for dying neonates, cross-cultural comparison of EoL practices is important, when NICU outcomes between countries are compared. There are no universal standards for treating neonates at EoL, and literature reveals these variations, with few studies examining EoL protocols. The extent to which physicians’ personal values and attitudes are associated with their practices of EoLDs remains to be clarified.

## Conclusions

In the present study, we emphasized on differences in existing EoL practices on a national basis. We evaluated physicians’ rationale for EoLDs and found that attitude directly associates to decision-making, or otherwise attitude influences practices. Finally, the attitude score was affected mainly by religiousness and position on the existing legal framework. A nationwide policy on EoL decisions along with transition to palliative care programs for incurable neonates should commence ethical, legal and professional discussions.

## Supplementary information

**Additional file 1:** Ethics Questionnaire.

**Additional file 2:** Results of univariate and multivariate analysis.

## Data Availability

Data are available upon a reasonable request from the corresponding author: Dr. Ilias Chatziioannidis e-mail: drilias@windowslive.com.

## Abbreviations

CPR: Cardiopulmonary resuscitation
CI: Confidence interval
EoLDs: End of life decisions
NICU: Neonatal Intensive Care Unit
Q25–Q75: Quantile 25–75
WH: Withhold
SD: Standard deviation
WD: Withdraw
