# Supplementary material for: Physicians’ attitudes in relation to end-of-life decisions in Neonatal Intensive Care Units: a national multicenter survey
Source: BMC Med Ethics. 2020 Nov 23;21:121. doi: 10.1186/s12910-020-00555-6 (PMC7681959; doi:10.1186/s12910-020-00555-6)
Supplement: Supplementary file 1 — Additional file 1: Ethics Questionnaire. [file 12910_2020_555_MOESM1_ESM.doc]

Aim of the present study is to investigate the ethical dilemmas of healthcare professionals who work in NICUs, along with the policy of their units concerning end-of-life care and parental participation in critical decision-making.

We thank you in advance for participating in the study and for your time.

**Nicoletta Iacovidou, MD, PhD**

Associate Professor of Paediatrics- Neonatology

Director of Neonatal Department, National and Kapodistrian University of Athens

Aretaieio Hospital, Athens, Greece

**Chatziioannidis Ilias**

Consultant in Neonatology and Paediatris

2^nd^ Neonatal Department and Neonatal Intensive Care Unit

**E Gkiougi**

Pédiatre Néonatologue

Conseillère DirectionMédicale CHR Liège

Belgium

**T Vidalis**

National Bioethics Commission

**Date ____ /____ /______**

**Ethics Questionnaire in NICUs**

**Demographic data**

| 1. **Gender** | **Male** |  | **Female** |  |  |
| --- | --- | --- | --- | --- | --- |

**2. Age**

**3. Having had children**

|  |
| --- |

| **4.** | **Religious Backround** | **Christian Orthodox**  **Catholic**  **Protestant** |  | **Muslim** |  | **Atheist/Agnostic** |  |
| --- | --- | --- | --- | --- | --- | --- | --- |

Country of origin: .............

**5. Importance of religion**

| Important |  |  |
| --- | --- | --- |
| Quite important |  |  |
| Not important |  |  |

| **6. Working Hours/week in the NICU** |  |
| --- | --- |

| **7.** | **Do you work every day in the NICU** | **Yes** |  | **No** |  |
| --- | --- | --- | --- | --- | --- |

**8. Rank**

| **for Doctors** | |  | **for Nurses** | |
| --- | --- | --- | --- | --- |
|  |  |  |  |  |
|  | Resident |  |  | Nurse |
|  | Fellow Resident |  |  | Midwife |
|  | Consultant Neonatologist |  |  | Nurse in charge |
|  | Research associate |  |  | Head Nurse |
|  | Neonatologist (part time) |  |  |  |

| **9. Years of employment in the NICU** | **/** | (years/months) |
| --- | --- | --- |

**10. Educational Level 12. Type of employment in the NICU**

|  | Graduate of Lower Technological Institute |  |  | Permanent |
| --- | --- | --- | --- | --- |
|  | High Technological Institute |  |  | Not permanent |
|  | University |  |  | Other ________________________________ |
|  | MSc |  |  |  |
|  | PhD |  |  |  |

| **11.** | **Partcipation on NICU’s Follow up Programs** | **Yes** |  | **No** |  |
| --- | --- | --- | --- | --- | --- |

| **13.** | **Participation in research protocols (the last 2 years)** | **Yes, often** |  | **No** |  |
| --- | --- | --- | --- | --- | --- |

**=====================================================================================**

**14. In the course of your professional practice in NICU, have you ever decided to limit treatment of a neonate:**

|  | -with poor neurologic prognosis | **Yes** |  | **No** |  |
| --- | --- | --- | --- | --- | --- |
|  | - at terminal stage | **Yes** |  | **No** |  |
|  | - with congenital syndrome with poor prognosis | **Yes** |  | **No** |  |
|  | -of extreme prematurity at the limit of viability | **Yes** |  | **No** |  |

**15. Regarding previous cases, when therapeutic interventions delay death, there are options as:**

- **withholding treatment**
- **withdrawing treatment**

**In the course of your professional practice in neonatal intensive care, which of the following actions you have decided or would have decided:**

| Withhold intensive care (resuscitation at birth, mechanical ventilation ) |  |
| --- | --- |
| Withhold emergency treatment (CPR) |  |
| Continue current treatment without adding further interventions |  |
| Withdraw mechanical ventilation |  |
| Administer sedatives/analgesics to alleviate pain even at the risk of respiratory depression and death |  |
| Administer drugs aiming at ending life |  |

**======================================================================================**

| **16.** | **What is your position on the existing legal framework ***  **Do you believe it should change** | **Yes** |  | **No** |  | **Not relevant** |  |
| --- | --- | --- | --- | --- | --- | --- | --- |

***** In Greece, euthanasia is strictly prohibited in accordance to Article 300 of Penal Code and Article 29 of the Code of Medical Ethics. Otherwise there is no existing legal framework on treatment limitation or palliative care.

**===================================================================================**

**17.** Please encircle the number that corresponds to your opinion:

| **1** | **2** | **3** | **4** | **5** |
| --- | --- | --- | --- | --- |
| **I strongly agree** | **I agree** | **I neither agree nor disagree** | **I disagree** | **I strongly disagree** |

| 1) As life is sacred, all actions should be taken in order to ensure neonatal survival regardless the severity of the prognosis | 1 | 2 | 3 | 4 | 5 |
| --- | --- | --- | --- | --- | --- |
| 2) Survival with severe physical handicap is preferable to life loss | 1 | 2 | 3 | 4 | 5 |
| 3) Survival with severe mental handicap is preferable to life loss | 1 | 2 | 3 | 4 | 5 |
| 4) Establishing policies of limited intensive care in critically ill neonates may lead to overuse in less severe cases | 1 | 2 | 3 | 4 | 5 |
| 5) Intensive care may lead to aggressive therapeutic interventions | 1 | 2 | 3 | 4 | 5 |
| 6) The family burden due to a disabled child should not be taken into account when  deciding whether or not to provide intensive care to a severely ill neonate | 1 | 2 | 3 | 4 | 5 |
| 7)There is no option for ethical decisions when there is no legal framework for limitation of treatment | 1 | 2 | 3 | 4 | 5 |
| 8) Every neonate should be provided with maximum intensive care irrespective of outcome, as the clinical experience acquired, will be beneficial to future patients | 1 | 2 | 3 | 4 | 5 |
| 9)The increasing cost of health care for preterm neonates and for disabled children limits the financial resources for maximized care of all neonates regardless the outcome | 1 | 2 | 3 | 4 | 5 |
| 10) From an ethical viewpoint, there is no differentiation between withholding and withdrawing of intensive care | 1 | 2 | 3 | 4 | 5 |
| 11) From the ethical point of view, there is no differentiation between withdrawal of intensive care and active euthanasia | 1 | 2 | 3 | 4 | 5 |
| 12) Withholding intensive care without taking active measures to end life could lead to severe disability if the neonate survives | 1 | 2 | 3 | 4 | 5 |

**Thank you for your participation and your time**

**Consent**

I consent for participation in the study.

I am aware it is strictly for research purposes

Signature
