# Supplementary material for: Physicians’ attitudes in relation to end-of-life decisions in Neonatal Intensive Care Units: a national multicenter survey
Source: BMC Med Ethics. 2020 Nov 23;21:121. doi: 10.1186/s12910-020-00555-6 (PMC7681959; doi:10.1186/s12910-020-00555-6)
Supplement: Supplementary file 2 — Additional file 2: Results of univariate and multivariate analysis. [file 12910_2020_555_MOESM2_ESM.docx]

**Supplement 2**

|  | **Univariate (N=160)** | | | **Multivariate (N=111)** | |
| --- | --- | --- | --- | --- | --- |
|  | **N** | **Attitude score (95%CI)** | **p** | **Result** | **p** |
| **Gender** |  |  | 0.5057 | Not included | |
| Female | 126 | 5.8 (5.4 - 6.2) |  |  |  |
| Male | 30 | 5.6 (4.8 - 6.5) |  |  |  |
| **Age** |  |  | 0.9782 | Not included | |
| ≥40 | 111 | 5.8 (5.4 - 6.2) |  |  |  |
| <40 | 45 | 5.7 (5.1 - 6.3) |  |  |  |
| **Having had children (parenthood)** |  |  | 0.3256 | Not included | |
| Yes | 109 | 5.8 (5.4 - 6.3) |  |  |  |
| No | 33 | 5.6 (5 - 6.3) |  |  |  |
| **Religion Importance** |  |  | **0.0240** | **0.6±0.4 units decrease of the attitude score for an opinion that religion is important** | **0.0989** |
| **Quite or not important** | **82** | **6.2 (5.7 - 6.6)** |  |  |  |
| **Important** | **71** | **5.3 (4.8 - 5.9)** |  |  |  |
| **Position on existing legal framework** |  |  | **0.0003** | **1.5±0.4 units increase of the attitude score for an opinion that the law framework requires reform** | **0.0022** |
| Should not change or is not relevant | 38 | 4.7 (4 - 5.4) |  |  |  |
| Should change | 104 | 6.3 (5.9 - 6.7) |  |  |  |
| **Education level** |  |  | **0.0973** | 0.5±0.4 units increase of the attitude score for MSc or PhD | 0.1889 |
| **MSc and/or PhD** | **69** | **6.1 (5.6 - 6.7)** |  |  |  |
| **Graduate from medical school** | **86** | **5.5 (5 - 5.9)** |  |  |  |
| **Involvement in research (at least one project in the last year)** |  |  | **0.0939** | 0.3±0.4 units increase for involvement n research | 0.4216 |
| **No** | **73** | **5.5 (5 - 5.9)** |  |  |  |
| **Yes** | **81** | **6.1 (5.5 - 6.6)** |  |  |  |
| **Physician’s Rank** |  |  | 0.1415 | Not included | |
| Specialized neonatologist | 89 | 5.6 (5.2 - 6.1) |  |  |  |
| Fellow | 58 | 6.2 (5.7 - 6.8) |  |  |  |
| **Working years in the NICU** |  |  | 0.6049 | Not included | |
| <15 | 115 | 5.7 (5.4 - 6.1) |  |  |  |
| ≥15 | 41 | 5.8 (5 - 6.7) |  |  |  |
| **Daily Duties in the NICU** |  |  | 0.5603 | Not included | |
| No | 39 | 5.9 (5.2 - 6.6) |  |  |  |
| Yes | 112 | 5.7 (5.2 - 6.1) |  |  |  |
| **Working hours in the NICU** |  |  | 0.6291 | Not included | |
| <40 | 38 | 5.7 (4.9 - 6.5) |  |  |  |
| ≥40 | 118 | 5.8 (5.4 - 6.2) |  |  |  |
| **Employment contract with the NICU** |  |  | 0.4802 | Not included | |
| Tenure | 82 | 5.9 (5.4 - 6.4) |  |  |  |
| Non-permanent | 69 | 5.7 (5.2 - 6.2) |  |  |  |
| **Follow up in outpatient clinic** |  |  | 0.6489 | Not included | |
| No | 32 | 5.9 (5 - 6.8) |  |  |  |
| Yes | 121 | 5.7 (5.3 - 6.1) |  |  |  |

Results of univariate and multivariate analysis for the role of the studied parameters in the attitude score. In the multivariate model were entered parameters that had p<0.1 in the univariate model. Parameters with p<0.1 are in bold. The number of cases included in the multivariate model are reduced (N=111) due to accumulation of missing entries, compared to the univariate approach (N from 142 to 156 depending on non-answered questions).
